# The Presence of Microplastics in the Gastrointestinal Tracts of Song Thrushes (*Turdus philomelos*) Wintering in Apulia (Southern Italy)—Preliminary Results

**DOI:** 10.3390/ani14142050

**Published:** 2024-07-12

**Authors:** Simona Tarricone, Maria Antonietta Colonna, Pierangelo Freschi, Carlo Cosentino, Giuseppe La Gioia, Claudia Carbonara, Marco Ragni

**Affiliations:** 1 Department of Soil, Plant and Food Sciences, University of Bari ‘Aldo Moro’, 70126 Bari, Italy; simona.tarricone@uniba.it (S.T.); mariaantonietta.colonna@uniba.it (M.A.C.); marco.ragni@uniba.it (M.R.); 2 School of Agricultural, Forestry, Food and Environmental Sciences (SAFE), University of Basilicata, 85100 Potenza, Italy; pierangelo.freschi@unibas.it (P.F.); carlo.cosentino@unibas.it (C.C.); 3 Or.Me., Via Saponaro 7, 73100 Lecce, Italy; ormepuglia@gmail.com

**Keywords:** microplastics, thrushes, plastic pollution, environmental quality, flotation

## Abstract

**Simple Summary:**

In recent decades, the use of plastic has increased exponentially. If discarded improperly, plastic waste can harm the environment and biodiversity. Under the influence of solar UV radiation, wind, currents, and other natural factors, plastic breaks down into small particles known as microplastics (MPs, <5 mm in one dimension) or nanoplastics (NPs, <100 nm). These tiny particles, which are characterized by various forms such as fragments, sheets, filaments, foam, granules, and pellets, have become a major factor in environmental pollution. Birds play an important role in the global trophic network system and are widely used as indicators of biodiversity, pollution, and environmental change. The present study aims to investigate the number of microplastics bioaccumulated in specimens of Song Thrushes (*Turdus philomelos*), which are migratory birds wintering in Apulia, Italy. The specimens (n = 360) were hunted in the Bari countryside and donated for research purposes by hunters. Plastic debris was detected in the stomachs of 129 and 128 birds shot in December and January. The majority of ingested MPs were fibers, followed by films, fragments, and pellets. As for color, among all the MPs found, 31.75% were red, 30.13% were black, and 25.91% were blue, while the other colors were less represented. The high contamination of thrushes confirmed the ubiquity of MPs in terrestrial ecosystems.

**Abstract:**

The term microplastics (MPs) describes a heterogeneous mixture of particles that can vary in size, color, and shape. Once released into the environment, MPs have various toxicological and physical effects on wildlife. The Song Thrush (*Turdus philomelos*) is a migratory species, staying in Italy in late autumn and winter. The aim of this study is to assess, quantify, and characterize the presence of microplastics in Song Thrushes hunted in the Apulia region of Italy. The birds (n = 360) were hunted in the Bari countryside and donated for research purposes by hunters. MPs were classified in relation to their shape in fibers, films, fragments, and pellets; then, they were divided according to their color and the length of the particles was measured. Nikon image analysis software was applied to the litter size measurements. Of the total of 360 birds, MPs were detected in the stomachs of 129 birds shot in December and 128 birds shot in January. The majority of ingested MPs were fibers that were observed in all contaminated birds. Film fragments were observed in every contaminated specimen. Among all the MPs found, 31.75% were red, 30.13% were black, and 25.91% were blue, while the other colors were less represented. This study provides the first analysis of MPs bioaccumulation in Song Thrushes wintering in the Apulia region, and the high contamination of thrushes confirmed the ubiquity of MPs in terrestrial ecosystems.

## 1. Introduction

Plastics are among the materials most used worldwide because of their excellent performance. In the last 15 years, despite various plastic reduction policy initiatives, the production of plastic has increased from 2 million tons (Mt) in 1950 to 367 Mt in 2020 [1], of which 58 million tons were produced in Europe [2]. The huge production of this synthetic organic polymer, which is made from petroleum, is due to its numerous properties, which are useful for a wide variety of applications in daily life, such as packaging, buildings, households, furniture, textiles, sports equipment, vehicles, electronics, and agriculture [3]. On the other hand, being poorly biodegradable, plastic is a very powerful source of pollution that threatens the safety and quality of food, human health, and coastal tourism and contributes to climate change. If discarded improperly, plastic waste can harm the environment and biodiversity. It has been estimated that about 14 million tons of plastic end up in the seas every year [4,5]. Plastic debris and waste represent a major concern for marine ecosystems and shorelines on all continents, with higher concentrations near crowded tourist destinations and densely populated areas. Under the influence of solar UV radiation, wind, currents, and other natural factors, plastic breaks down into small particles known as microplastics (MPs, <5 mm one dimension) or nanoplastics (NPs, <100 nm), which gradually accumulate in the environment [6]. The term MPs describes a heterogeneous mixture of particles that can vary in size (from a few microns to several millimeters), color, and shape (from very different shapes of fragments to long filaments). Once released into the environment, MPs have various toxicological and physical effects on wildlife. Several chemicals used in the production of plastics are known to be carcinogenic and interfere with the body’s endocrine system, leading to developmental, reproductive, neurological, and immune disorders in humans and wildlife [7,8,9]. In addition, MPs can restrict the movement of animals [10], and when ingested, MPs can damage and clog the gastrointestinal tract, which can lead to reduced food intake, starvation, and direct mortality [11,12,13,14].

In past years, most research on MPs has been conducted in aquatic environments and organisms [15,16,17,18], but contamination of terrestrial ecosystems is thought to be much greater [19]. Birds play an important role in the global food web and thrushes, given their terrestrial lifestyle and the wide distribution of many species, have been used as a potential indicator of MP pollution in terrestrial environments [20]. Previous studies on other terrestrial birds have shown that many of the harmful effects arise from the ingestion of plastic particles dispersed in the environment or from the ingestion of prey (through trophic transfer) that have previously been exposed to pollutants [21].

Thrushes *(Turdus* sp.) are important game species in the western and central Mediterranean countries, with annual bags comprising tens of millions of birds [22]. In Italy, 96% of all ring recoveries are due to human activities, predominantly hunting [23]. The Song Thrush (*Turdus philomelos*) is among the most representative game bird species in Italy [24,25], migrating over short to medium distances [26], and present in Southern Italy in late autumn and winter [27,28]. They might have evolved as forest birds, but, currently, they can be observed in both closed forests and open habitats and are non-adapted to life in wetlands [29,30]. They forage mainly from the ground and search for prey in leaf litter, grass, and moss [20]. Thrushes usually feed on invertebrates (insects, snails, and worms), as well as fruits and seeds. Due to their widespread occurrence and terrestrial habitat, thrushes may be a potential indicator of MP pollution in various landscapes with different degrees of urbanization.

Therefore, the aim of this study is to investigate whether Song Thrushes caught in the Apulian territory during the hunting season could have ingested microplastics, in order to quantify and characterize them.

## 2. Materials and Methods

### 2.1. Sampling

**Figure 1:**
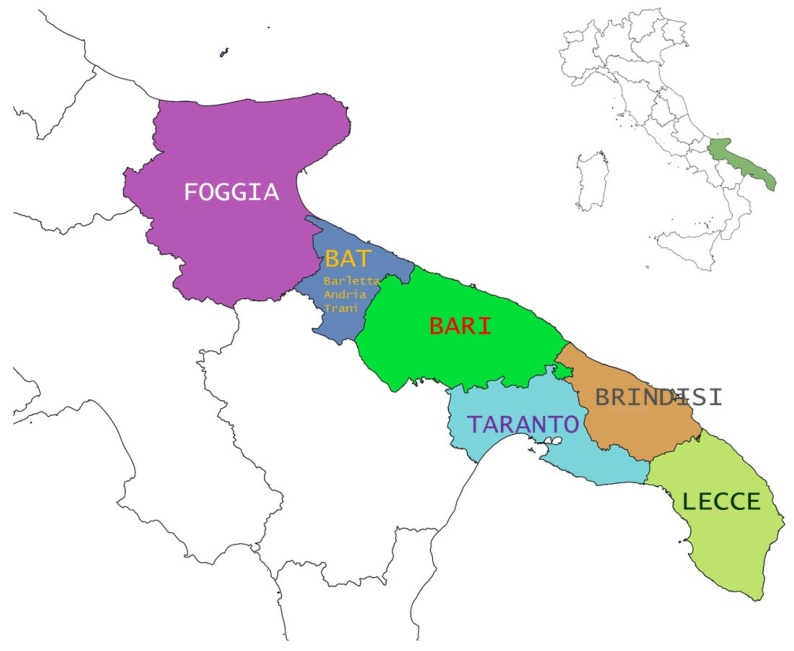
Geographic representation of the Apulia Region.

For the trial, 360 Song Thrushes (*Turdus philomelos*) were used, which were hunted in the provinces of Bari, BAT, and Lecce (Apulia region, Italy; Figure 1). The researchers who participated in the experiment were not involved in the hunting of the animals; the local hunters, authorized by the Apulian region, provided 60 dead birds for the objectives of this research every ten days during December and January, without any other purpose. With the DGR n 1058/2022 [31] and subsequent amendments, the Apulia Region has allowed hunting of the Song Thrush until 29 January 2023, as this species in Apulia is not yet in the breeding season or returning to the breeding grounds at this time, as shown in previous reports [32,33,34,35].

### 2.2. Contamination Prevention and MPs Extraction

In accordance with the guidelines recommended by Prata et al. [30], we followed quality and control procedures in all our experiments. Specifically, we used only glass and metal materials, including glass centrifuge tubes, and wore cotton lab coats; all equipment, containers, and beakers were rinsed three times with filtered distilled water before and after use and covered with aluminum foil to prevent airborne microplastic contamination. All fluids (saline solution and distilled water) were filtered with a cellulose nitrate filter membrane with a pore size of 1 µm and a diameter of 47 mm [36] (Axiva Sichem Biotech, Delhi, India) before use. At the same time, a blank extraction sample without tissue was performed to detect and correct any contamination from the process.

The carcasses were placed in individual cotton bags, frozen within 12 h of collection, and stored at −20 °C until dissection and analysis. A necropsy was performed on each bird to extract the entire stomach. The thrush stomachs were put into a beaker with 100 mL of fixative solution (70% ethanol) for five minutes to prevent infection risk; subsequentially, the stomachs were chopped, and the mixture obtained was divided into three beakers. Each sample was filtered through a filter paper (Whatman 1, with particle retention >11 µm) and the 3 g of the residual part was added to 10 mL of NaCl solution 1.8 g/cm^3^. The higher density of this solution proved to be the most effective method for separating all the microplastics from the matrix. All the glass tubes were centrifuged at 1500 rpm for 3 min. After centrifugation, a small amount of NaCl solution was added to each tube to obtain the typical meniscus of the flotation method. A cover slip was placed on the meniscus to capture the supernatant and load it onto a microscope slide.

### 2.3. Preparation, Quantification, and Characterization of MPs Flotation

After 10 min, the slides were observed under a stereomicroscope (Nikon, Tokyo, Japan) to analyze the presence of potential plastic particles, and the images were captured with a digital camera (Nikon X_Entry, Tokyo, Japan) [36].

MPs—795 in December and 600 in January—were classified as fibers, films, fragments, and pellets according to the guidelines of Rochman et al. [6]. The colors were classified as black, brown, blue, silver, green, gray, red, white, and transparent; the length of the detected particles was determined, and each particle was assigned to one of three distinct size classes: 50–100 µm, 101–500 µm, or 501–1000 µm. Nikon image analysis software (Nikon X_Entry, Tokyo, Japan) was applied to the litter size measurements [36].

Each MP, after the description, was subjected to a hot needle test, as described by other authors [37,38,39], which has already been shown to be reliable for detecting MPs larger than 50 µm.

### 2.4. Statistical Analysis

The parameters were analyzed using a two-factor ANOVA model. The factors were defined as follows: (1) number of MPs per plastic-containing bird (Factor 1: month; factor 2: size of MPs); (2) color incidence (%) (Factor 1: month; Factor 2: type of color); (3) MPs size incidence (%) (Factor 1: month; Factor 2: fiber length). With regards to the percentage data, in order to test the differences among the incidence (%) of the four types of MPs, we first converted the percentages to proportions (values between 0 and 1) and, successively, the following angular transformation was applied: θ = arcsin ($\sqrt{p}$), where p is the proportion. To better understand the magnitude of these differences, we back-transformed the mean angular values to their original percentage scale. Means were compared by Tukey’s HSD. Significance was declared at *p* < 0.05. Data were analyzed using SAS software 9.1 2004 [40].

## 3. Results

Of the total 360 Song Thrushes (*Turdus philomelos*), plastic debris was detected in the stomachs of 129 (71.67%) and 128 birds (71.11%) shot in December and January, respectively (Table 1). The contamination from airborne microplastics in the blank sample was very low, with an average value of 0.28; the plastic debris found in the blank samples was eliminated on all counts.

A total of 795 and 600 MPs were found in birds caught in December and January, respectively. Table 2 shows the number of MPs per plastic-containing bird. A significant difference in the type of MPs was found for fibers in birds caught in December (*p* < 0.05). Birds hunted in December showed a higher number of fragments and pellets, but a lower number of films.

**Figure 2:**
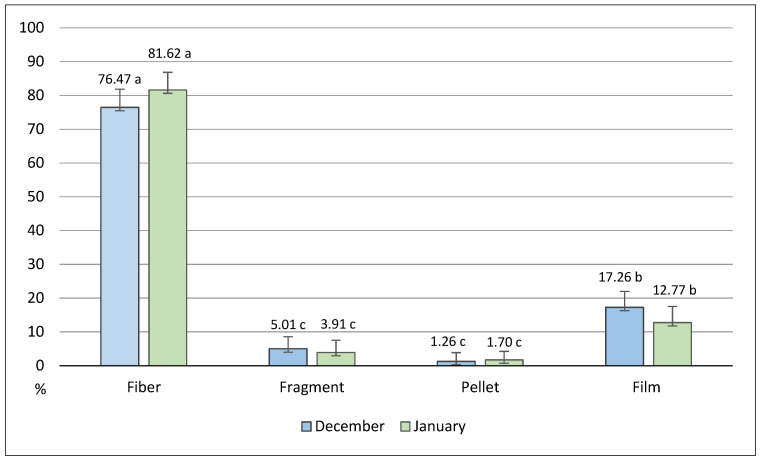
Percentage of each type of microplastic, calculated on the total of MPs detected in December and January. Different letters indicate differences for *p* < 0.05.

Figure 2 shows the percentage of each type of microplastic found in the plastic-containing birds. No significant differences between months were aroused for any type of MP. Fibers were the most frequently recorded, with significant (*p* < 0.05) differences in comparison to all the other types, followed by films, whose percentage was also markedly (*p* < 0.05) greater as compared to fragments and pellets.

**Figure 3:**
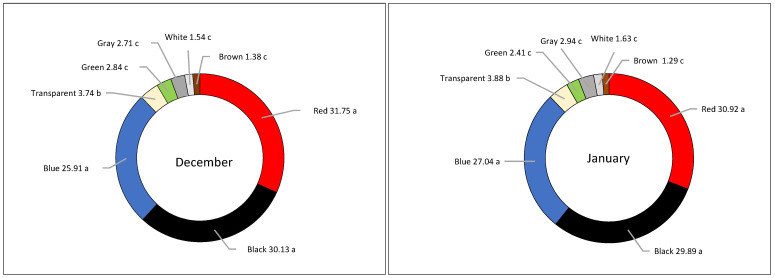
Percentage of MPs colors in the stomachs of Song Thrushes (% of each color in the total number of MPs) in December and January. Different letters indicate differences for *p* < 0.05.

No significant differences between months were found for the colors of microplastics. As shown in Figure 3, the MPs found in the plastic-containing birds showed different colors which were, in order of frequency, as follows: red, black, blue, transparent, gray, green, and white. On average, MPs were red (31.75 vs. 30.92%), black (30.13 vs. 29.89%), and blue (25.91 vs. 27.04%), respectively, in December and January, whereas the other colors were less represented.

**Figure 4:**
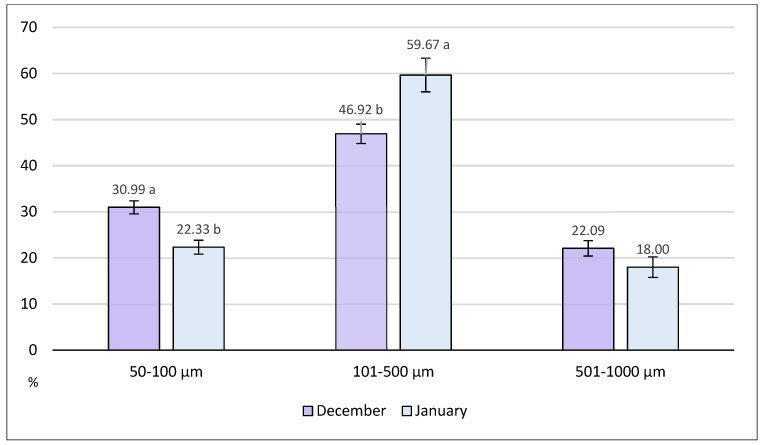
Percentage distribution of microplastics (mean ± SE) in relation to the length class (within classes: a, b *p* < 0.05).

Figure 4 shows the distribution of microplastics in relation to three distinct length classes: 50–100, 101–500, and 501–1000 µm. The most representative class of length was the intermediate one, with particles ranging from 101 to 500 µm. Birds hunted in December had a significantly higher percentage of particles smaller than 100 µm (*p* < 0.05), while thrushes caught in January showed a higher incidence of particles within the length range of 101–500 µm (*p* < 0.05). No significant differences between months were found for this parameter.

**Figure 5:**
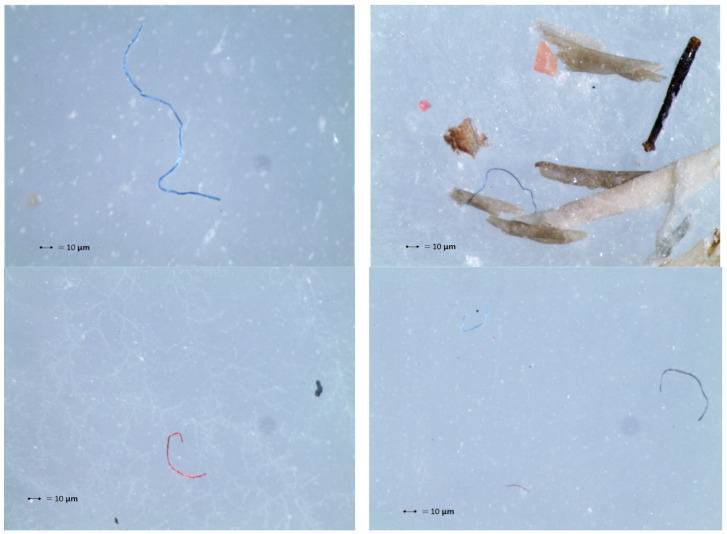
Stereomicroscope images showing different types of microplastics found in the Song Thrushes’ stomachs.

**Figure 6:**
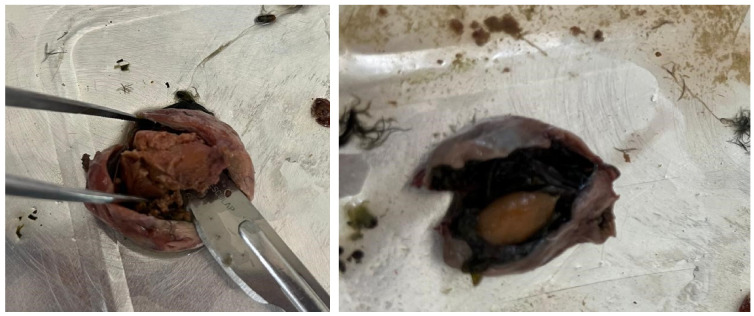
Olive seeds found in the Song Thrushes’ stomachs.

Figure 5 shows the stereomicroscope images capturing the different types of microplastics found in the Song Thrushes’ stomachs, with details on the type and color of microplastics. Figure 6 shows details of photos of olive seeds swallowed by the birds and the presence of the whole olive stone in the stomach.

## 4. Discussion

In this trial, we investigated for the first time the occurrence of MPs in the stomach of Song Thrushes hunted in the Apulia Region, South Italy (Figure 5). Of the total 1395 plastic particles found during the study, fibers dominated with 988 pieces (70.83%), followed by MP film (20.83%), MP fragments (5.83%), and MP pellets (2.37%). The number of MPs found in the stomachs of thrushes hunted in Apulia was lower than the number of MPs detected in Song Thrushes from Poland by Deoniziak et al. [20]. Also, compared to other migratory bird species, the number of MPs detected in Apulian Song Thrushes is lower [41,42]. Zhao et al. [43] analyzed the digestive tracts of 12 different bird species, including the gray-backed thrush (*Turdus hortulorum*), detecting anthropogenic waste in all specimens. According to their results, opportunistic and omnivorous species, which have a variable diet, show an increased risk of MP ingestion through prey (secondary ingestion). Other authors [42,44,45] have indicated that birds ingest microfibers, but do not retain them like other microplastics, as they find them in feces; despite this, MP fibers were the most representative particles in our study.

Fibers were also more abundant than fragments in a study conducted in China [43]; the authors hypothesized two reasons for the high presence of fibers. First, the smaller overall size of fibers folded, knotted, or twisted into an aggregate may increase their likelihood of being ingested. Indeed, the fibers observed were often inextricably twisted. Second, the ubiquity of fibers in clothing, furniture, feminine hygiene products, and diapers could make them more bioavailable.

Also, in the study of Specchiulli et al. [46], the predominant forms of MPs in Lesina Lagoon (Apulian region) have been identified as filaments and fragments, suggesting that the majority of MPs in the lagoon are of secondary origin [47]. Polypropylene (PP) and polyethylene (PE) were among the most detected polymers, used mainly as packaging materials, which explains their widespread occurrence in the Lesina Lagoon [46].

The color of MPs has become an important research topic, as many animal species are visual predators and can selectively forage for prey based on color [48,49]. Colors may be associated with the original polymer components or added to the polymers during the manufacturing process. Over time, these colors may fade or change due to environmental weathering [49]. In our analysis, we found that the red color was the major representative (31.75%), followed by black (30.13%) and blue (25.91%); the other colors were less represented. Zhao et al. [43] analyzed the stomachs of terrestrial birds and found that colored particles were more abundant than light or dark ones. The dominance of mid-tone particles (red, blue, etc.) may be the result of their prevalence in the environment, easy detection, or their resemblance to food, resulting in an actual color preference by the biota at lower trophic levels [50]. In contrast, for Carlin et al. [51], blue- and clear-colored fibers were the most common colors observed in aquatic birds and they suggested three possibilities that could explain why these color patterns were observed. First, the dominant colors found in the animals were also the dominant colors of MPs in the landscapes where the subjects were foraging. Blue and clear were the dominant colors of MPs in the water column in estuaries, bays, and marine waters in many global studies, and birds can drink water containing MPs [52]. Second, birds may grab macro- or microplastics that are clear or blue-colored when foraging or nesting, mistaking them for suitable prey or nesting materials [53,54]. Third, the prey of these apex predators might preferentially, or accidentally, consume blue or clear microplastics [55]. These possibilities are not mutually exclusive but may be linked and occur simultaneously. According to our results, plastic coloring increases the attractiveness of the product [56], but it also resembles food, increasing the likelihood of being ingested [57,58]. Song Thrushes are terrestrial birds that, in the Apulia region, commonly live in woods and olive groves, feeding on berries and invertebrates. They may confuse berries and olives which are red and black with MP particles [59]. Furthermore, MPs may be ingested by feeding on insects. Arnold et al. [60] suggested that MPs can be transferred to insectivore birds; indeed some researchers detected MPs in several types of insects [61,62,63,64,65].

The largest part of MPs observed during this study were smaller than 1 mm and 80% of them were smaller than 500 µm, as demonstrated by other authors [20,66]. Zhen et al. [67] divided the length of the particles into three smaller classes: 20–50 µm, 50–100 µm, and 100–500 µm, and reported that more than 70% of particles found in the terrestrial birds fell within the 20–50 µm range.

This may reflect the ability of omnivorous birds to ingest a wide variety of anthropogenic debris that can accumulate in their stomachs. Although we did not directly assess trophic transfer in this study, our findings of microparticles in the stomachs of thrushes suggest that this mid-trophic species, as insectivorous predators and prey, likely contribute to the trophic transfer of microparticles. A possible hypothesis, supported by the discovery of whole olives in the thrushes’ stomachs (Figure 6), is that the greater incidence of microplastics in December may be linked to the greater ingestion of olives during this month since, in Apulia, the period from October to December is characterized by intense activity linked to the harvesting of ripe olives to produce extra virgin olive oil. Whether the greater presence of workers and production of plastic wastes during this period could have affected a higher presence of MPs in song thrushes’ stomachs, or the fact that the birds swallowed olives along with fragments of the plastic nets commonly placed under the trees for harvesting olives, are issues that must be confirmed by future research.

Terrestrial microscopic anthropogenic particles are a type of pollution that is “close to home” and deserves more public attention. The analysis of the MPs in the bioindicators aids in assessing the potential MPs pollution of the environment, considering that MPs can act as vectors for pathogens and chemical pollutants because of their environmental persistence and potential ecotoxicity, which pose significant concerns for human health [68,69]. However, MPs’ potential risks for human health are still unclear, and should not be ignored [70]. Microscopic anthropogenic litter and its associated complex of chemical contaminants have the potential to transfer to wildlife. Previous studies have reported that the ingestion of microplastic debris can affect overall fitness in birds [70,71]. Golubev has analyzed the death of some penguin chicks, attributing the cause to macroplastics present in the stomach [72]. Plastics may provoke several potential adverse effects due to their role in the release and transport of environmental chemical contaminants [73]. Roman et al. [74] reported that chemicals from microplastics ingested by Japanese quail (*Coturnix japonica*) resulted in minor delays in chick sexual maturity and growth, but not in adult body weight.

## 5. Conclusions

This study represents the first investigation of the bioaccumulation of MPs in Song Thrushes wintering in Apulia and makes a contribution to the understanding of MP exposure in terrestrial wildlife in Italy and Europe. The significant contamination observed in thrush populations emphasizes the ubiquitous presence of MPs in terrestrial ecosystems. Subsequent studies should prioritize the comprehensive investigation of the entire gastrointestinal tract and extend sampling to thrushes from wintering and nesting habitats. The use of birds that died from collisions with anthropogenic structures as a non-invasive method of collecting material for analysis is recommended. In addition, future research efforts should include multiple sites, cover different time periods, and conduct thorough MP assessments of the environment to fully utilize the potential of the Song Thrush as a bioindicator.

## Figures and Tables

**Table 1:** Number of Song Thrushes with presence of microplastics in the stomach.

Hunting Months	December	January
Number of plastic-containing birds	129	128
Number of plastic-free birds	51	52

**Table 2:** Number of microplastics per plastic-containing bird.

Type of Microplastic	December		January		*p*-Value
	Mean ± SE	Birds, n	Mean ± SE	Birds, n	
Fiber	3.04 ± 0.125	129	2.63 ± 0.123	128	0.021
Fragment	1.81 ± 0.291	31	1.12 ± 0.286	33	0.091
Pellet	1.33 ± 0.548	9	1.12 ± 0.399	17	0.751
Film	1.95 ± 0.178	86	2.33 ± 0.228	52	0.197

## Data Availability

Data are contained within the article.
